# A rare case of extensive biventricular cardiac sarcoidosis with reversible torrential tricuspid regurgitation

**DOI:** 10.1007/s12350-023-03307-2

**Published:** 2023-05-31

**Authors:** Joseph Okafor, Alessia Azzu, Raheel Ahmed, Barbara Cassimon, Kshama Wechalekar, Athol Wells, Vasileios Kouranos, A. John Baksi, Rakesh Sharma, Kaushik Guha, Rajdeep Khattar

**Affiliations:** 1 Department of Echocardiography, Royal Brompton & Harefield Hospitals, Guy’s and St, Thomas’ NHS Foundation Trust, London, UK; 2 National Heart & Lung Institute, Imperial College London, Guy Scadding Building, Dovehouse St, London, SW3 6LY UK; 3 Cardiovascular Magnetic Resonance Unit, Royal Brompton & Harefield Hospitals, Guy’s and St. Thomas’ NHS Foundation Trust, London, UK; 4 Cardiac Sarcoidosis Service, Royal Brompton & Harefield Hospitals, Guy’s and St, Thomas’ NHS Foundation Trust, London, UK; 5 Department of Nuclear Medicine and PET, Royal Brompton & Harefield Hospitals, Guy’s and St. Thomas’ NHS Foundation Trust, London, UK; 6 Department of Cardiology, Portsmouth Hospitals University Trust, Portsmouth, UK

**Keywords:** Sarcoid heart disease, PET, echo, MRI, valvular heart disease, amyloid heart disease

## Abstract

Reversal of torrential tricuspid regurgitation is rarely seen. We describe a case in which effective immunosuppression alongside conventional heart failure therapies lead to reversibility of torrential tricuspid regurgitation in a patient with cardiac sarcoidosis. We also discuss the diagnostic challenge in distinguishing cardiac sarcoidosis from other myocardial diseases in a patient presenting with biventricular failure.

## History of presentation

A 48-year-old man presented with a 2-week history of worsening exertional dyspnea, abdominal distension, and fatigue. His past medical history included hypertension, Gilbert’s syndrome, pulmonary embolism in 2014 and 2020 for which he was taking apixaban and candesartan. Physical examination revealed a heart rate of 88 bpm, BP of 96/67 mmHg, raised JVP, mild ascites, and bilateral leg edema. A pan-systolic murmur was audible and air entry at the right lung base was reduced. 12-lead electrocardiogram demonstrated sinus rhythm with 1st degree atrioventricular block (AVB), PR interval 273 ms, right bundle branch block with QRS duration 131 ms, and left axis deviation. Inpatient rhythm monitoring detected episodes of second degree (Mobitz II) AVB. Echocardiography revealed severe impairment of biventricular systolic function.

## Investigations

Serologic work-up revealed polycythemia (Hb 167 g·L^−1^, PCV 0.51) with normal inflammatory markers (WCC 3.7 × 10^9^·L^−1^, CRP 3 mg·L^−1^), mild renal impairment (urea 10.2 mmol·L^−1^, creatinine 113 μmol·L^−1^), and elevated bilirubin (56 μmol·L^−1^). Liver transaminases, corrected calcium, and thyroid function tests were normal. High-sensitivity troponin-I (40 ng·L^−1^, < 19.8), brain natriuretic peptide (5655 ng·L^−1^, 0-20), and serum angiotensin converting enzyme (104 U·L^−1^, 13-64) were elevated. Tuberculosis Elispot test was negative. Despite the polycythemia, molecular diagnostics detected no mutations in *JAK2, CALR*, and *MPL* genes.

**Figure 1:**
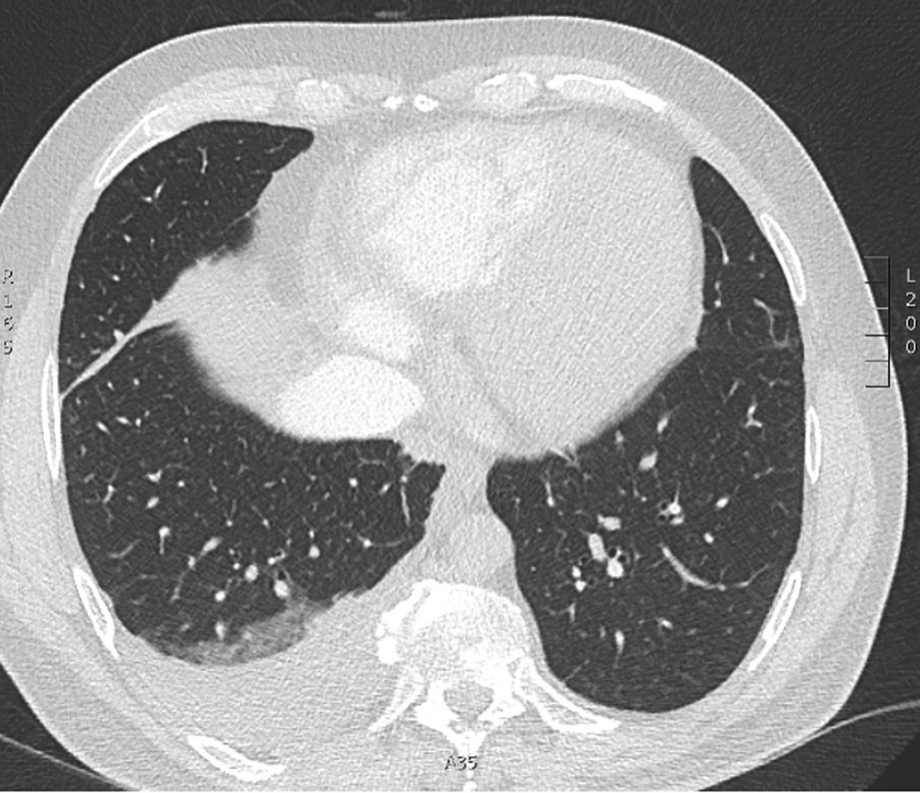
Computed tomography of the thorax. Lung parenchymal nodules and nodal enlargement in keeping with sarcoidosis. Cardiomegaly and right-sided pleural effusion secondary to decompensated cardiac failure

Computed tomography of the thorax excluded a pulmonary embolus but demonstrated peribronchovascular lung nodules with an upper lobe predominance and multiple enlarged hilar lymph nodes, typical of sarcoidosis (Figure 1).

Echocardiography revealed a markedly dilated left ventricle (LV) with severely reduced ejection fraction (EF) of 14% and multiple regional wall motion abnormalities, not compatible with a coronary distribution. Moderate functional mitral regurgitation (MR) was noted. The myocardium had a speckled appearance especially of the right side of the interventricular septum. The right ventricle (RV) was dilated with severely reduced systolic function (fractional area change [FAC] 6%, 3D RVEF 20%). There was non-coaptation of the tricuspid valve (TV) leaflets due to tricuspid annular dilatation causing torrential tricuspid regurgitation (TR) and a dilated inferior vena cava. Because of the severe RV dysfunction and torrential TR, the RV systolic pressure could not be estimated.

**Figure 2:**
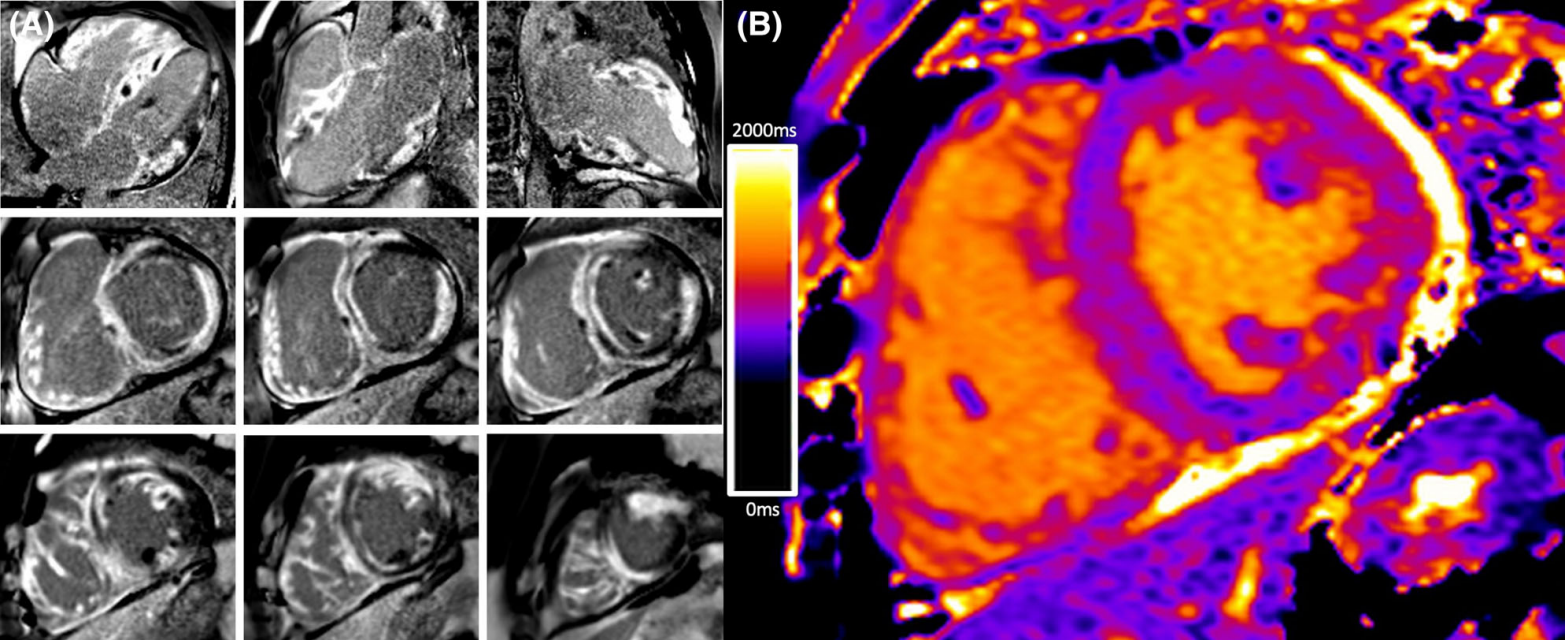
Cardiac magnetic resonance imaging. (**A**) Extensive biventricular delayed enhancement involving the right ventricle trabeculae. “Zebra-like” enhancement of the interventricular septum is often seen in cardiac amyloidosis. (**B**) Increased native myocardial T1 values in the mid-lateral wall

Cardiac magnetic resonance (CMR) imaging confirmed severe biventricular dysfunction with no regions of T2 signal-hyperintensity. Delayed gadolinium imaging revealed near circumferential subendocardial and subepicardial enhancement in the septum, anterior, and inferior walls which became transmural in the basal-mid lateral wall (Figure 2). This appearance on CMR accompanied by the speckled myocardial echotexture on echocardiography raised the possibility of cardiac amyloidosis (CA). The RV free wall and proximal inter-atrial septum displayed diffuse enhancement. The mid-lateral wall and apical septum showed increased native myocardial T1 values (~ 1100 ms at 1.5 T).

**Figure 3:**
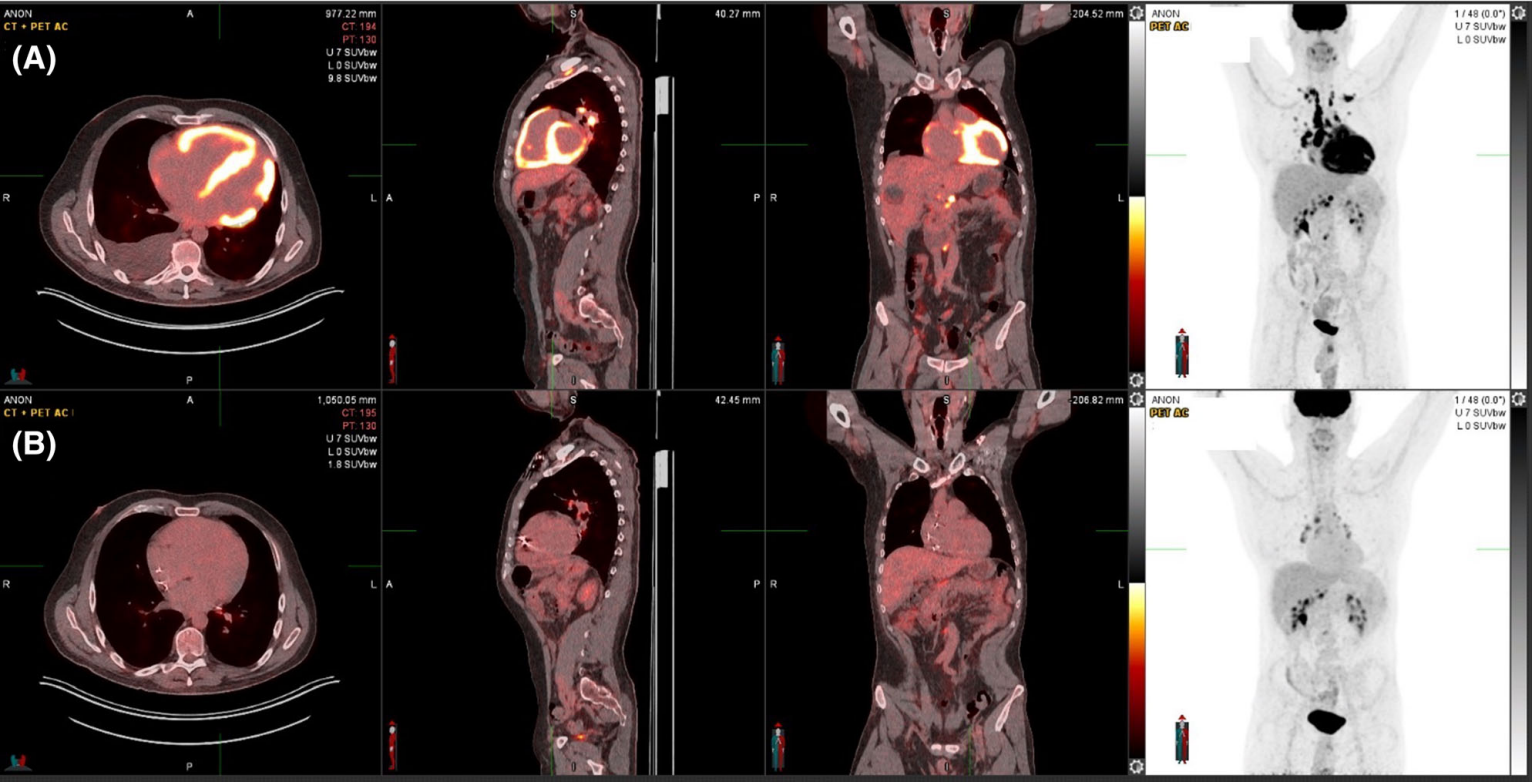
Response to immunosuppressive therapy. FDG-PET at baseline (**A**) with intense myocardial inflammation sparing only parts of the lateral wall and apex and 6 months after treatment (**B**). FDG-PET = 18F-fludeoxyglucose positron emission tomography

**Figure 4:**
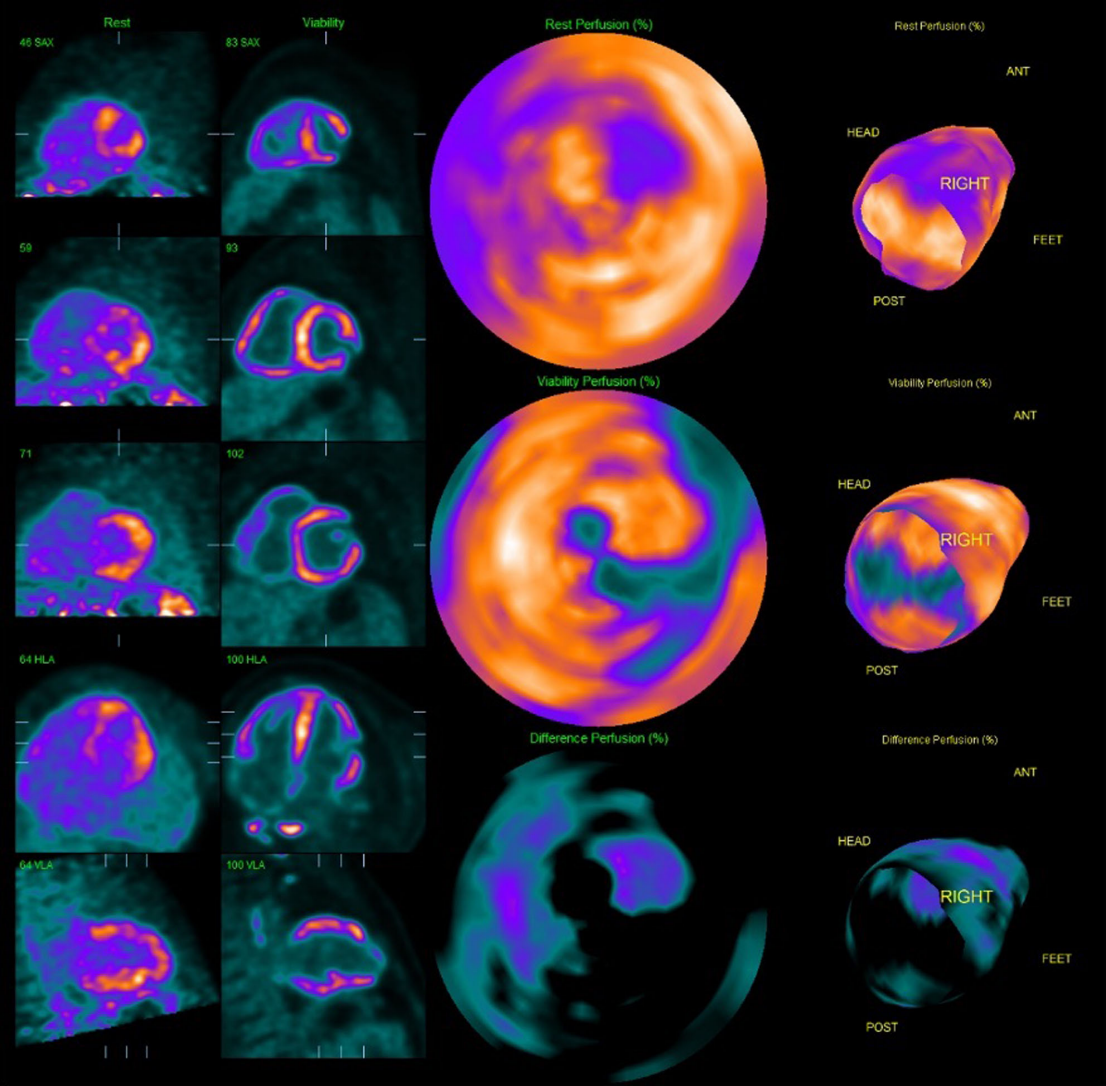
Perfusion and metabolism. Mismatch pattern typical of intensively active sarcoidosis

**Figure 5:**
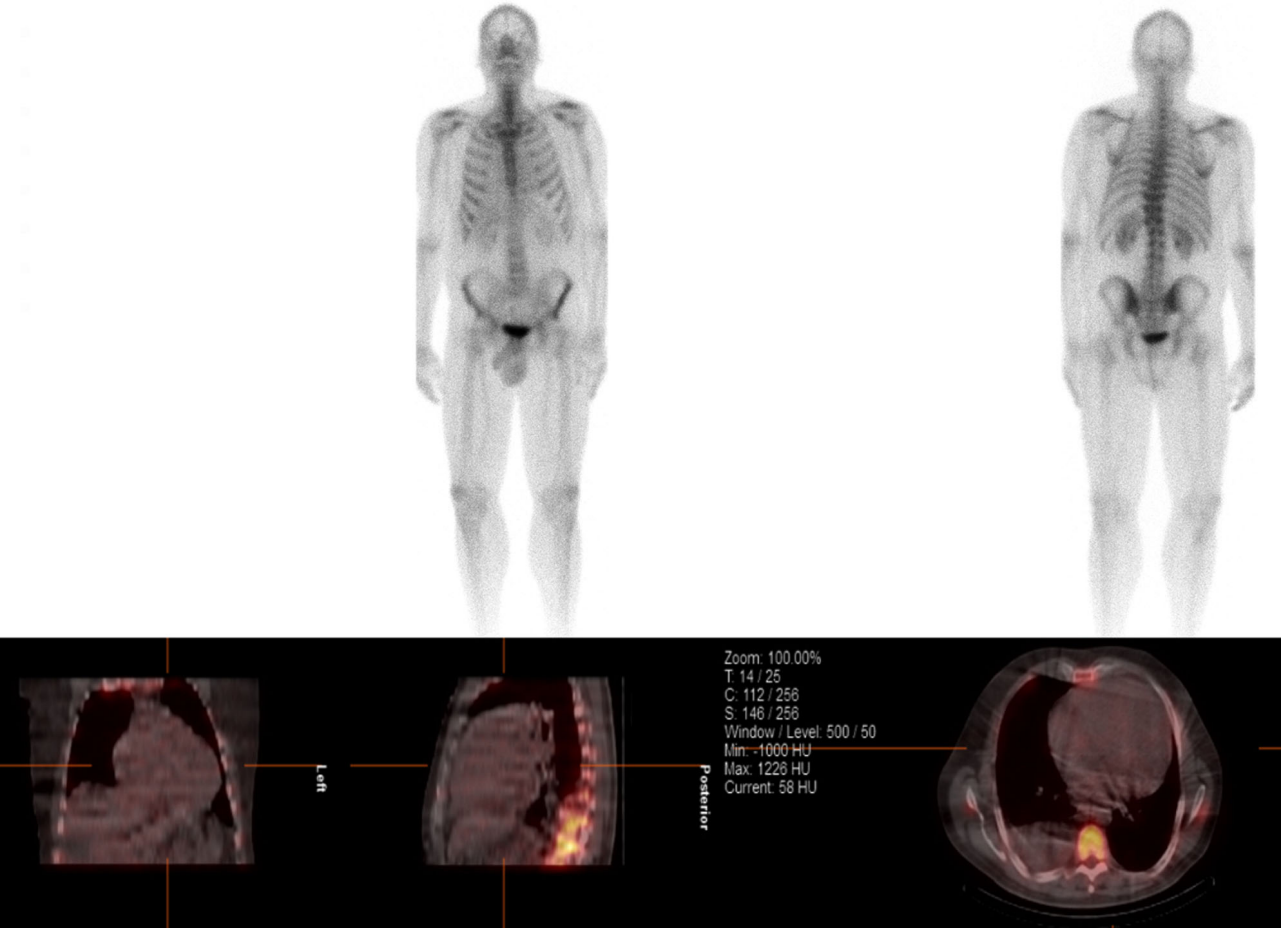
Technetium 99m (Tc-99m)-DPD and Single-photon emission computed tomography (SPECT). Absence of the bone tracer uptake within the myocardium hence exclusion of transthyretin (ATTR type) amyloidosis. *Note* Negative Tc-99m DPD imaging does not exclude AL amyloidosis

Subsequent 18F-fludeoxyglucose (FDG) positron emission tomography (PET) revealed intense thoracic and abdominal lymphadenitis with a maximal standardized uptake value (SUV_max_) of 14. Patchy intense FDG activity was seen throughout the myocardium (SUV_max_ 11.9) involving the septum, anterior, inferior, and RV free walls, both atria and the inter-atrial septum (Figure 3A). Multi-territory perfusion-metabolism mismatch was noted in a pattern typical of active CS (Figure 4). Advanced inflammatory and perfusion quantification was undertaken on co-localized Rubidium-82 and FDG-PET/CT images. This revealed significant inflammatory extent comprising 88% of the LV myocardium and a perfusion defect extent of 47%. Mean SUV of the whole myocardium was 6.7. Rare possibility for CA to produce a myocardial inflammatory signal was considered but the high intensity was not in favor of CA. In addition, a normal Tc-99m-DPD and SPECT/CT scan excluded transthyretin amyloidosis (Figure 5) while a normal serum amyloid P component (SAP) scan showed no amyloid deposits. Serum analysis showed no detectable M protein or free light chains, rendering AL amyloidosis highly unlikely. After multidisciplinary discussion endomyocardial biopsy and histological examination of the RV septum was performed revealing extensive fibrosis containing scattered multinucleated giant cells and non-necrotizing granulomas, confirming the diagnosis of CS. Congo red staining was negative for amyloidosis.

## Management

The fluid overload resolved with aggressive intravenous diuresis alongside oral heart failure medications. The patient was treated with induction therapy using intravenous methylprednisolone infusions (1 g on 3 consecutive days). Following this, prednisolone (40 mg daily weaned to 15 mg over 10 weeks) and methotrexate (7.5 mg gradually increased to 20 mg weekly) were initiated. Due to episodes of high-grade AVB and severe LV systolic impairment, a defibrillator with cardiac resynchronization therapy (CRT-D) was implanted. Device follow-up at 6 weeks demonstrated 97% biventricular pacing with the underlying QRS duration 135 ms. Six-months later, repeat FDG-PET imaging revealed almost complete resolution of myocardial inflammation with only low-grade FDG uptake in the basal septum (SUV_max_ 2.7). The hilar lymph nodes remained mildly enlarged and inflamed with a reduction in SUV_max_ to 6.5 (Figure 3B**)**.

Echocardiography at 6 months demonstrated improvement in LVEF to 33%. The RV remained dilated but FAC improved to 16%. Remarkably there was marked reduction in TR to a moderate grade, with clear leaflet coaptation despite the new RV pacing lead in situ, and PASP was 16 mmHg. At 15 months, there was sustained improvement in biventricular function (LVEF 44%, 3D RVEF 36%, FAC 35%) and the TR remained moderate.

## Discussion

TV incompetence in CS is rare and usually secondary to chamber dilatation. Direct valvular granulomatous infiltration was described in a case following cardiac transplantation.^1^ The patient had severe TR prior to transplantation and histopathology revealed non-caseating granulomas within the TV leaflets, moderator band, RV septum, and the right atrium. Extensive fibrosis of the TV causing adherence to the RV wall was responsible for the valvular regurgitation. In our case, primary myocardial involvement of the RV likely resulted in chamber and TV annular dilation, although it is possible that concomitant TV leaflet and papillary muscle inflammation may have contributed to malcoaptation. Reduction in TR was therefore felt to be predominantly due to immunosuppressive therapy, as the improvement in RV function and TV leaflet coaptation corresponded with resolution of RV inflammation on FDG-PET. However, CRT implantation also played an important role in promoting LV remodeling.

In 90% of cases the most frequent mechanism of TR is secondary rather than primary. De novo TR following device implantation is associated with recurrent HF hospitalization and increased mortality.^2^ Other causes include left-sided valvular and myocardial disease, pulmonary hypertension and increased RV afterload from pulmonic stenosis.^3^ Regurgitation occurs due to TV annular dilation, leaflet tethering, and RV +/− right atrial remodeling. Therefore, treatment of the primary cause often improves TR. Reversibility of torrential TR in the presence of severe RV dilatation and dysfunction, as in our case, has rarely been described. Reversible severe TR has been noted in association with thyrotoxicosis, wherein dilatation of the RV cavity and TV annulus was ascribed to a hyperdynamic circulatory state. Following beta-blockade and restoration of euthyroid status, only trivial TR was detectable.^4^ In the majority of cases, however, TR-reversibility is seen in the context of medical therapy for HF and atrial fibrillation whereby chronic right atrial dilatation is the core mechanism.

Distinguishing between cardiac sarcoidosis and amyloidosis was a clinical challenge. When advanced, both diseases may display biventricular infiltration manifesting as granular speckling and thickening of the myocardium on echocardiography.^5^ Key echocardiographic differentiators include thinning and aneurysmal transformation of the RV free wall or interventricular septum in CS compared to hypertrophy in CA.^6^ Diffuse thickening of the interatrial septum or cardiac valves may also be seen in CA. With CMR, LGE in a patchy, multifocal distribution is common to both diseases. In our case, the global subendocardial LGE pattern that is a hallmark of CA, was present in addition to transmural and subepicardial fibrosis. Expansion of the interstitial space with abnormal amyloid protein accumulation causes increased extracellular volumes. An elevated native T1 relaxation time > 1164 ms yields a 98% positive predictive value for CA.^7^ However, myocardial inflammation on FDG-PET, in particular the high signal with the RV free wall, tilted the balance toward CS. Due to differences in likelihoods on advanced imaging findings, endomyocardial biopsy was performed and, in this case, the histology clarified the diagnosis.

## New knowledge gained

This was a clinical case with several learning points including understanding of the mechanisms of valvular disease in cardiac sarcoidosis. We discussed how prompt immunosuppression combined with medical and device-related heart failure therapy can aid reversal in severe tricuspid regurgitation. Finally, we discussed the importance of multimodality imaging in distinguishing between cardiac sarcoidosis and amyloidosis.

## Abbreviations

AVB: Atrioventricular block
CA: Cardiac amyloidosis
CMR: Cardiac magnetic resonance
CS: Cardiac sarcoidosis
EF: Ejection fraction
FDG-PET: 18F-fludeoxyglucose positron emission tomography
LV: Left ventricle
RV: Right ventricle
TR: Tricuspid regurgitation
TV: Tricuspid valve
